# A Cohesive Framework for Motor Stereotypy in Typical and Atypical Development: The Role of Sensorimotor Integration

**DOI:** 10.3389/fnint.2017.00019

**Published:** 2017-08-25

**Authors:** Robin L. Shafer, Karl M. Newell, Mark H. Lewis, James W. Bodfish

**Affiliations:** ^1^Vanderbilt Neuroscience Graduate Program, Vanderbilt Brain Institute, Vanderbilt University Nashville, TN, United States; ^2^Department of Kinesiology, University of Georgia Athens, GA, United States; ^3^Department of Psychiatry, University of Florida Gainesville, FL, United States; ^4^Department of Hearing and Speech Sciences, Vanderbilt University Medical Center Nashville, TN, United States

**Keywords:** repetitive behavior, complexity, entropy, motor development, goal-directed behavior, autism

## Abstract

Stereotyped motor behavior manifests as rhythmic, repetitive movements. It is common in several neurologic and psychiatric disorders where it is considered maladaptive. However, it also occurs early in typical development where it serves an adaptive function in the development of complex, controlled motor behavior. Currently, no framework accounts for both adaptive and maladaptive forms of motor stereotypy. We propose a conceptual model that implicates sensorimotor mechanisms in the phenomenology of adaptive and maladaptive stereotypy. The extensive structural and functional connectivity between sensory and motor neural circuits evidences the importance of sensory integration in the production of controlled movement. In support of our model, motor stereotypy in normative development occurs when the sensory and motor brain regions are immature and the infant has limited sensory and motor experience. With maturation and experience, complex movements develop and replace simple, stereotyped movements. This developmental increase in motor complexity depends on the availability of sensory feedback indicating that the integration of sensory information with ongoing movement allows individuals to adaptively cater their movements to the environmental context. In atypical development, altered neural function of sensorimotor circuitry may limit an individual’s ability to integrate sensory feedback to adapt movements to appropriately respond to environmental conditions. Consequently, the motor repertoire would remain relatively simple, resulting in the persistence of motor stereotypy. A framework that considers motor stereotypy as a manifestation of low motor complexity resulting from poor sensorimotor integration has many implications for research, identification and treatment of motor stereotypy in a variety of developmental disorders.

## Introduction

Stereotyped motor behavior is traditionally defined as rhythmic, repetitive, invariant movement. It occurs in a vast number of species ranging from invertebrates such as worms and insects to vertebrates such as birds and mammals—including humans (Thelen, 1979; Garner et al., 2003; Lewis and Kim, 2009; Stephens et al., 2011; Berman et al., 2014). Despite its ubiquity, there is a conflict surrounding the phenomenon of motor stereotypy. It can be adaptive, as it is in healthy human infants, where it is a transitional state in motor development. It can also be maladaptive, as it is in a variety of neurodevelopmental, neuropsychiatric and neurologic disorders, where it interferes with goal-directed behavior. Research on motor stereotypy and the conceptual and neurobiological models aimed at understanding its genesis focus on either the adaptive or the maladaptive aspects of the behavior. Currently, there is no framework that accounts for both manifestations. Here we present a mechanistic framework that accounts for both the adaptive and maladaptive presentation of motor stereotypy.

## Stereotypy as a Transitional State in Healthy Motor Development

Stereotyped motor behaviors occur in early infancy as a transitional state of motor development. Thelen (1979) observed that simple, repetitive behaviors including arm waving and body rocking preceded complex motor behaviors—goal-directed reaching and crawling, respectively—that involved the use of the same body segments. In a study of infant repetitive kicking, Thelen and Fisher (1983) measured electromyography and joint-angle rotation in infants’ legs. At around 1 month of age, kicks were characterized by tight temporal and spatial synchrony of the hip, knee and ankle joints and a simultaneous contraction of antagonistic muscle groups during the flexion phase followed by passive movement during the extension phase. Similar muscle activation and joint angle relationships occur during supported stepping in 1 month old infants (Thelen and Cooke, 1987). By 2 months of age, the ankle rotation is less correlated with the knee and hip joints, eventually reaching an adult-like negative correlation by the time infants are walking independently. Additionally, complex, phasic muscle activation replaces simple, tonic co-contraction of the muscles.

A similar transition occurs in the arm when infants are learning how to reach for a toy. Before they are able to execute controlled, accurate reaches, infants generate repetitive arm movements with patterns of motor activity that are inefficient relative to the dynamic physical properties of the arm and the goal of reaching the toy (Thelen et al., 1993; Konczak et al., 1995). Using these inefficient reach approximations as a starting point, infants explore the dynamics of their arms through adjusting the amplitude and timing of muscle activation, ultimately allowing them to generate more accurate and efficient reaches with muscle activation patterns that more closely resemble adult-like patterns. These findings demonstrate that simple, stereotyped motor behavior in infants is the foundation on which complex, functional behavior is built. However, this adaptive view of motor stereotypy does not account for stereotyped behavior in clinical conditions.

## Stereotypy in Developmental, Neurologic and Psychiatric Disorders

Stereotyped motor behavior is present in a variety of developmental, neurologic and psychiatric disorders including fronto-temportal dementia (Mendez et al., 2005), schizophrenia (Morrens et al., 2006) and neurodevelopmental disorders (NDD), including autism spectrum disorder (ASD; Goldman et al., 2009; American Psychatric Association, 2013). It can be induced in animals via lesions (e.g., ventromedial thalamic nucleus (Zainos et al., 1984), nigrostriatal dopamine projections (Simola et al., 2007)), pharmacological agents, genetic manipulations, or barren cage environments (Lewis et al., 2007; Stearns et al., 2007; Peça et al., 2011). In clinical populations, motor stereotypy is considered abnormal, maladaptive and apparently purposeless (Cooper and Dourish, 1990) as it is not goal-directed. Unlike in normative development, stereotypy in disease states poses a functional impairment by interfering with complex, adaptive behavior. For example, stereotypy in individuals with ASD has been shown to interfere with play (Koegel et al., 1974) and learning (Koegel and Covert, 1972; Morrison and Rosales-Ruiz, 1997).

Because motor stereotypy is highly prevalent in disease, it is often indicative of neural pathology. It is primarily associated with deficits in cortico-striatal-thalamic circuitry. Parkinson’s disease patients have degeneration of dopaminergic medium spiny neurons that project to the striatum (Braak and Del Tredici, 2008). Lesioning these dopaminergic projections in animals recapitulates many symptoms of Parkinson’s disease including tremor and levadopa induced dyskinesia (Simola et al., 2007). Additionally, injecting dopamine agonists into the striatum induces stereotypy in rodents (Kelley et al., 1988; Delfs and Kelley, 1990). Animal studies of autism associated genes (Stearns et al., 2007; Peça et al., 2011) and cage-induced stereotypy (Presti and Lewis, 2005; Tanimura et al., 2010) also implicate alterations of basal ganglia circuitry in the phenomenology of stereotyped motor behavior. However, neurologic disorder or insult does not account for motor stereotypy in healthy infant development.

## A Coherent Framework for Stereotypy in Typical and Atypical Development

***Hypothesis**: poor sensorimotor integration results in low motor complexity leading to the presence of stereotyped behavior in both normative development and disease states*.

Motor complexity provides an adaptive advantage for interacting with the environment since higher complexity permits more flexibility in motor output. This is based on Bernstein’s (1967) concept of skill as a reflection of mastering redundant degrees of freedom in motor learning. He posited that the body is composed of multiple biomechanical degrees of freedom that can be utilized in several ways to achieve the same goal. Motor control is contingent on the ability to use these degrees of freedom to flexibly interact with the environment. In normative development, when the motor system is immature or in a state of early motor learning, the system constrains the degrees of freedom to gain some control and produce stable movements for a given task. This results in simple movements such as the repetitive kicking that Thelen and Fisher (1983) observed. As the motor system matures or learning progresses, degrees of freedom are released to permit greater specificity and efficiency of movement. This is supported by findings that higher motor complexity is associated with more accurate motor task performance (Deutsch and Newell, 2001; Mosconi et al., 2015).

Our model elaborates on Bernstein’s (1967) postulate by emphasizing an important role of sensorimotor integration in the ability to release biomechanical degrees of freedom to produce controlled, complex movements. Sensorimotor integration involves communication between the sensory and motor systems in the brain allowing for: (a) the use of sensory input to generate an accurate and efficient motor plan (e.g., through an inverse model); and (b) the use of self-generated and external sensory feedback to monitor and correct error in the movement (e.g., updating the forward model in the ongoing movement or for future movements; see (Wolpert et al., 1998) for a description of these processes in the cerebellum). Under our framework, sensory input allows the motor system to plan and execute accurate movements by making optimal use of the available degrees of freedom and to correct error in the movement by exploiting the available degrees of freedom to make adjustments that are appropriate to the error.

In the case of stereotyped behavior in healthy infants, our model suggests that the infant brain does not efficiently integrate sensory information with the motor system because either the sensory and motor regions of the brain are immature, or the infant has limited experience with or access to sensory information (e.g., due to immobility). Poor sensorimotor integration prevents the infant from using his/her degrees of freedom flexibly and efficiently, limiting his movements to simple, stereotyped behaviors. With maturity, the infant is able to integrate sensory information with motor behavior permitting him to release degrees of freedom to produce complex movements.

Similarly, our model posits that deficits in sensorimotor integration contribute to the emergence and maintenance of stereotyped behavior in clinical disorders. Whether it is caused by atypical development of sensorimotor circuitry, degenerative processes, or another form of altered neural function, these alterations could contribute to the persistence of stereotyped behavior by disrupting the sensory inputs that are required to inform and diversify motor repertoires.

## Neural Circuitry Supporting Sensory Influence on Motor Function

**Figure 1:**
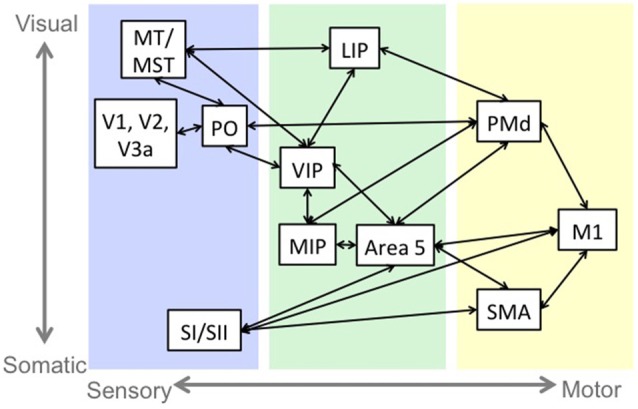
An example of sensorimotor circuitry: the reach pathway. Blue indicates regions that are primarily responsive to sensory stimulation. Green indicates regions that have sensory and motor responses, and yellow indicates regions that have primarily motor related activity. There is a tendency in the sensory and sensorimotor areas for regions near the top of the figure to represent visual information and the regions near the bottom to represent somatosensory information. Abbreviations: middle temporal area (MT), medial superior temporal area (MST), primary visual cortex (V1), secondary visual cortex (V2), visual area 3a (V3a), parieto-occipital area (PO), primary somatosensory cortex (SI), secondary somatosensory cortex (SII), lateral intraparietal cortex (LIP), medial intraparietal cortex (MIP), ventral intraparietal cortex (VIP), dorsal premotor cortex (PMd), primary motor cortex (M1), supplementary motor area (SMA).

The role of sensorimotor integration in motor control and complexity is consistent with the structural and functional connectivity of sensorimotor neural circuitry. While this sensorimotor connectivity occurs in several brain regions involved in motor control, including cortex, basal ganglia (Flaherty and Graybiel, 1994) and cerebellum (Wiestler et al., 2011; Proville et al., 2014), we will describe the cortical reach pathway, depicted in Figure 1, as it is a well-studied example of this phenomenon.

Visual information from prestriate (V2) and extrastriate (V3, V3a, MT/MST) cortices (Maunsell and van Essen, 1983; Colby et al., 1988; Lewis and Van Essen, 2000) and somatosensory information from primary (SI) and secondary (SII) somatosensory cortices (Jones and Powell, 1969; Pandya and Seltzer, 1982; Cipolloni and Pandya, 1999) projects to sensorimotor regions including the lateral (LIP), ventral (VIP) and medial (MIP) intraparietal areas and Brodmann’s area 5. SI and SII also project to frontal regions including primary motor cortex (M1), and supplementary motor area (SMA; Jones and Powell, 1969; Pandya and Seltzer, 1982; Cipolloni and Pandya, 1999). The intraparietal areas are interconnected (Blatt et al., 1990; Lewis and Van Essen, 2000), and represent target, eye and limb position in various reference frames (Gnadt and Andersen, 1988; Ferraina and Bianchi, 1994; Brotchie et al., 1995; Johnson et al., 1996; Duhamel et al., 1997; Pesaran et al., 2006; Bremner and Andersen, 2012).

The sensorimotor areas project to frontal motor regions including dorsal premotor cortex (PMd), SMA and M1 (Pandya and Kuypers, 1969; Jones and Powell, 1970; Jones et al., 1978; Strick and Kim, 1978; Jürgens, 1984; Petrides and Pandya, 1984; Johnson et al., 1996), which are active during motor planning (Alexander and Crutcher, 1990; Pesaran et al., 2006) and execution (Johnson et al., 1996). The motor cortices send the motor command to the body and to sensory and sensorimotor cortices to inform the sensorimotor system of the expected sensory consequences of the movement and monitor movement accuracy (Nelson, 1996; Desmurget and Grafton, 2000; MacDonald and Paus, 2003; Haggard and Whitford, 2004; Christensen et al., 2007; Mulliken et al., 2008).

Additional studies of the functional role of sensorimotor connectivity indicate that visual information influences the neural activity of an ongoing motor pattern (Mulliken et al., 2008; Archambault et al., 2009); voluntary movement induces activation in SI that is associated with PMC activity when proprioceptive input is blocked (Christensen et al., 2007), and the deactivation of the superior parietal lobule impairs the perception of visual-motor congruency for self-generated but not passive movements (MacDonald and Paus, 2003).

Our model suggests that poor functional integration in this circuitry may influence both the emergence of motor stereotypy in early typical development and its persistence in NDDs.

## Motor Complexity in Typical Development

In addition to Thelen and colleagues’ studies in typical infants, the development of motor complexity has been studied in other contexts. When infants are first learning to sit and can support their posture only briefly, their motor profile, as measured via center-of-pressure, is less complex than it is a few months later, when they are able to support their posture for extended periods of time (Harbourne and Stergiou, 2003). Similarly, the center-of-pressure profiles of young children while standing still are less complex than those of school-age children or young adults (Newell, 1998). A developmental pattern is also observed in the force exertion profiles for isometric grip force tasks (Deutsch and Newell, 2001, 2002, 2003; Smits-Engelsman et al., 2003) and the temporal structure of gait (Hausdorff et al., 1999) such that young children have less complex motor profiles than older children or adults. These findings demonstrate that motor complexity increases over the course of normative development.

## Sensory Influence on Motor Complexity in Typical Development

In keeping with the proposed model, there is evidence that sensory feedback can influence developmental changes in motor complexity. Thelen (1980) found that rates of stereotyped movements were inversely related to the amount of vestibular input (rocking, bouncing, swinging, etc.) provided by caregivers, and stereotypy persisted later in infants who received less vestibular input. The frequency of stereotypy also increased when movement was restricted (e.g., the infant was in a playpen, walker, or chair) compared to when the infant was allowed to move freely. Additionally, in pre-ambulatory infants, the rotation of the hip, knee and ankle become less coupled and resemble mature ambulation if the infants are supported while stepping on a treadmill (Thelen, 1986). The pull of the treadmill on the infants’ rear legs elicits stepping movements with more complex joint-angle relationships than the infants are able to generate independently at that stage.

Other paradigms have also elucidated developmental changes in motor complexity that depend on the sensory context. The double-step reaching task requires participants to reach to a visual target (Van Braeckel et al., 2007; Hyde and Wilson, 2013; Wilson and Hyde, 2013; Ruddock et al., 2015). On most of the trials, the target remains stationary, but on a subset of the trials, the target shifts mid-reach to a new location. This requires the participant to use continuous visual and proprioceptive feedback to efficiently alter the movement trajectory to accurately touch the target. Younger children are less efficient in correcting their movements when the target shifts than older children or adults. This developmental pattern is maintained when controlling for variables that measure motor planning and execution independently of the stimulus condition (e.g., reaction time, time to peak velocity).

Using a quantitative assessment of motor complexity, Deutsch and Newell (2001, 2002, 2003) measured approximate entropy and power spectral frequency of the force output during an isometric force task in children and adults. They found age-related increases in approximate entropy and frequency representation when the participants were provided with visual feedback of their force exertion. However, when visual feedback was removed, all age groups displayed relatively low complexity. These findings were replicated in a postural sway task (Newell, 1998), further supporting the contributions of age and sensory feedback to motor complexity.

## Motor Complexity in Neural Pathology

Motor complexity is atypical in several neurologic disorders that present with stereotypy (Sprague and Newell, 1996; Newell et al., 1999; Bodfish et al., 2001; Hong et al., 2006; Newell and Bodfish, 2007; Kent et al., 2012). Here, we will focus on the relation of motor complexity to stereotypy in NDDs. Most of this work has focused on adults with stereotyped body rocking (Newell et al., 1999; Bodfish et al., 2001; Hong et al., 2006; Newell and Bodfish, 2007). Newell et al. (1999) analyzed the position time series of joint position during body rocking using approximate entropy. Individuals with stereotyped body rocking had less complex joint position profiles than typically developing individuals. Similarly, individuals with stereotyped body-rocking displayed lower complexity in their center-of-pressure profiles than typically developing individuals when they were sitting still (Hong et al., 2006; Newell and Bodfish, 2007) or standing still (Bodfish et al., 2001) on a force platform. When participants engaged in body rocking, the typically developing participants reduced their motor complexity to the level of the individuals with stereotyped body rocking; whereas, the participants with stereotyped body rocking displayed low complexity in both conditions (Hong et al., 2006; Newell and Bodfish, 2007). These studies indicate that motor stereotypy is a manifestation of low motor complexity.

Consistent with these findings, Mosconi et al. (2015) observed that individuals with ASD (ages 5–35 years), had lower approximate entropy of sustained grip force, relative to age-matched, typically developing individuals. They also found a trend for typically developing individuals to show a greater age-related increase in motor complexity than individuals with ASD indicating that individuals with ASD have an abnormal developmental trajectory of motor complexity.

## Sensorimotor Integration in Developmental, Neurologic and Psychiatric Disorders

Many neurologic and psychiatric disorders that present with stereotypy have known sensorimotor deficits (Takarae et al., 2004; Quednow et al., 2008; Lencer et al., 2010; Morris et al., 2015; Nebel et al., 2015; Wang et al., 2015). Unfortunately, sensory abnormalities, motor deficits and stereotyped behaviors in these disorders have largely been studied in isolation and without regard for how they may relate to one another.

In ASD, for example, stereotyped behavior is diagnostic (American Psychatric Association, 2013), but motor deficits (Mostofsky et al., 2006; Duffield et al., 2013) and unusual sensory behaviors (Kwakye et al., 2011; Kirby et al., 2015; Stewart et al., 2016) are also highly prevalent and are some of the earliest symptoms (Sacrey et al., 2015). Few studies have found associations between stereotyped behavior and sensory, motor, or sensorimotor abnormalities (Boyd et al., 2009, 2010) underscoring the need for additional research exploring the relation between these symptom domains. Stereotyped behavior in ASD emerges in infancy as it does in typical development (Thelen, 1979; Kim and Lord, 2010), but unlike in typical development, stereotypy persists in individuals with ASD. While the literature in NDDs, including ASD supports the link between reduced motor complexity and the presence of motor stereotypy, there are currently no studies that assess the effect of sensory feedback on motor complexity in individuals with ASD or related NDDs. Given the importance of sensory feedback for motor complexity in typical development, we hypothesize that reduced motor complexity in individuals with ASD and related NDDs results from poor sensorimotor integration. However, additional research is needed to explore this hypothesis.

## Conclusions

Traditionally, motor stereotypy has been studied from two distinct perspectives: it can serve a functional role, as in normative development where it provides a foundation for the development of goal-directed behavior, or it can be maladaptive, as in neurologic and psychiatric disorders where it interferes with functional behavior. At present, there is no unifying framework to explain these two manifestations of motor stereotypy. We have introduced a model arguing that both healthy and pathologic forms of motor stereotypy manifest when motor complexity is low as a result of poor sensorimotor integration. This model is consistent with the established structure of sensorimotor neural circuitry. Support for this model comes from existing studies of stereotypy in typical development and NDDs that demonstrate a relation between stereotypy and motor complexity, as well as studies demonstrating the importance of sensory feedback for developmental increases in motor complexity in typical development.

There are several critical gaps in the research relating to early identification, treatment and etiology of conditions associated with stereotypy that can be considered in relation to the proposed model:

1. Early Risk Markers: diagnosing children with pathologic conditions that present with stereotypy often is not possible until after symptoms manifest; however, under our model, careful tracking of the development of motor complexity may be used to determine when at-risk infants veer from the normative trajectory. Early identification of at-risk infants permits earlier treatment interventions, which could prevent or minimize the expression of stereotypy in these individuals.
2. Early Intervention: if our model is accurate, interventions could aim to enhance motor complexity, for example by engagement with sensorimotor activities that require the child to vary his/her motor patterns. Treatment interventions that have enriched the home environments through exposure to various sensory and motor activities or have reinforced variability in behavior have successfully reduced repetitive behaviors in children with ASD (Boyd et al., 2011; Woo and Leon, 2013; Woo et al., 2015). Importantly, these studies did not test whether decreased stereotypy was mediated by increased motor complexity, but this should be explored in future studies.
3. Pathogenesis: motor complexity and sensorimotor integration can be examined reliably in clinical populations and in animal models. Adapting tasks used to assess the influence of sensory feedback on motor complexity in typical development (e.g., Deutsch and Newell, 2001, 2002, 2003) for use in individuals with ASD or other NDDs would provide additional support for our conceptual framework, while the use of animal models provides a means for studying the etiology of stereotypy and the effect of sensory feedback on motor complexity at levels of behavioral, neuronal and molecular analysis that are inaccessible in humans.

## Author Contributions

RLS, KMN, MHL and JWB were involved in the conceptualization of important intellectual content for the article, were involved in revising the work critically for important intellectual content, gave his or her final approval of this version of the article and agree to be accountable for all aspects of the work. RLS drafted the manuscript.

## Conflict of Interest Statement

The authors declare that the research was conducted in the absence of any commercial or financial relationships that could be construed as a potential conflict of interest.
